# An integrated single-cell transcriptomic dataset for non-small cell lung cancer

**DOI:** 10.1038/s41597-023-02074-6

**Published:** 2023-03-27

**Authors:** Karolina Hanna Prazanowska, Su Bin Lim

**Affiliations:** 1 Department of Biochemistry & Molecular Biology, Ajou University School of Medicine, Suwon, 16499 Korea; 2 Department of Biomedical Sciences, Ajou University Graduate School of Medicine, Suwon, 16499, Korea

**Keywords:** High-throughput screening, Data integration

## Abstract

As single-cell RNA sequencing (scRNA-seq) has emerged as a great tool for studying cellular heterogeneity within the past decade, the number of available scRNA-seq datasets also rapidly increased. However, reuse of such data is often problematic due to a small cohort size, limited cell types, and insufficient information on cell type classification. Here, we present a large integrated scRNA-seq dataset containing 224,611 cells from human primary non-small cell lung cancer (NSCLC) tumors. Using publicly available resources, we pre-processed and integrated seven independent scRNA-seq datasets using an anchor-based approach, with five datasets utilized as reference and the remaining two, as validation. We created two levels of annotation based on cell type-specific markers conserved across the datasets. To demonstrate usability of the integrated dataset, we created annotation predictions for the two validation datasets using our integrated reference. Additionally, we conducted a trajectory analysis on subsets of T cells and lung cancer cells. This integrated data may serve as a resource for studying NSCLC transcriptome at the single cell level.

## Introduction

The technology of whole-transcriptome single-cell RNA sequencing (scRNA-seq) was first introduced in 2009^1^. Since then, this technique has rapidly emerged as a powerful tool for studying cellular heterogeneity in various fields, including Oncology^2,3^. The number of publicly available scRNA-seq datasets containing samples from various tissues and species greatly increased within the past decade, with the National Center for Biotechnology Information (NCBI) Gene Expression Omnibus (GEO)^4,5^ being one of the most popular platforms dedicated to deposition of such data. However, small cohort size, inclusion of limited cell types, and insufficient annotation of cell populations are common obstacles to efficient reuse of the data, often slowing down the analysis. Therefore, several strategies have been developed for integration of the scRNA-seq data and correction of technical differences between the samples, also termed as batch effect^6^.

Among these strategies, Harmony^7^ and Seurat^8^ are commonly recommended^9,10^. Seurat identifies pairs of cells in a similar biological state across the datasets, termed anchors, and uses them to organize the data into a single integrated, corrected expression matrix. In this approach, cell subpopulations shared between different datasets are identified using canonical correlation analysis (CCA) and mutual nearest neighbours (MNNs)^11,12^. Seurat also enables data transfer between scRNA-seq datasets. In data transfer, principal component (PC) structure of a reference dataset is projected onto the query based on transfer anchors, and annotation predictions are generated for query cells^11^. In contrast to Seurat, Harmony integration operates on the PCs values, which represent a low-dimensional embedding of the original expression matrix and projects cells from different batches into a new shared embedding. Rather than using CCA, Harmony clusters cells in a way to obtain a balanced ratio of cells from different batches in each cluster, via k-means clustering and cluster centroid correction^10,13^. In our analysis, we decided to perform the integration and batch correction using Seurat.

Lung and bronchus cancer is the leading cause of cancer mortalities worldwide, with non-small cell lung cancer (NSCLC) accounting for the majority of new lung cancer cases^14,15^. Histologically, NSCLC is commonly classified as one of the two most common subtypes, including lung adenocarcinoma (LUAD) and squamous cell carcinoma (LUSC)^16^. LUAD has been confirmed to originate mostly from type two alveolar epithelial cells of the lung, whereas LUSC can arise either from basal cells of the bronchial epithelium, club cells, or alveolar cells^16^. Growing evidence suggests a prognostic and predictive value of diverse cell types in NSCLC, including fibroblasts, immune, and endothelial cells^17–19^. A detailed single-cell atlas exploring a variety of cell populations would thus provide insight into the tumor microenvironment and help unveil novel markers for improvement of NSCLC therapy.

**Fig. 1:**
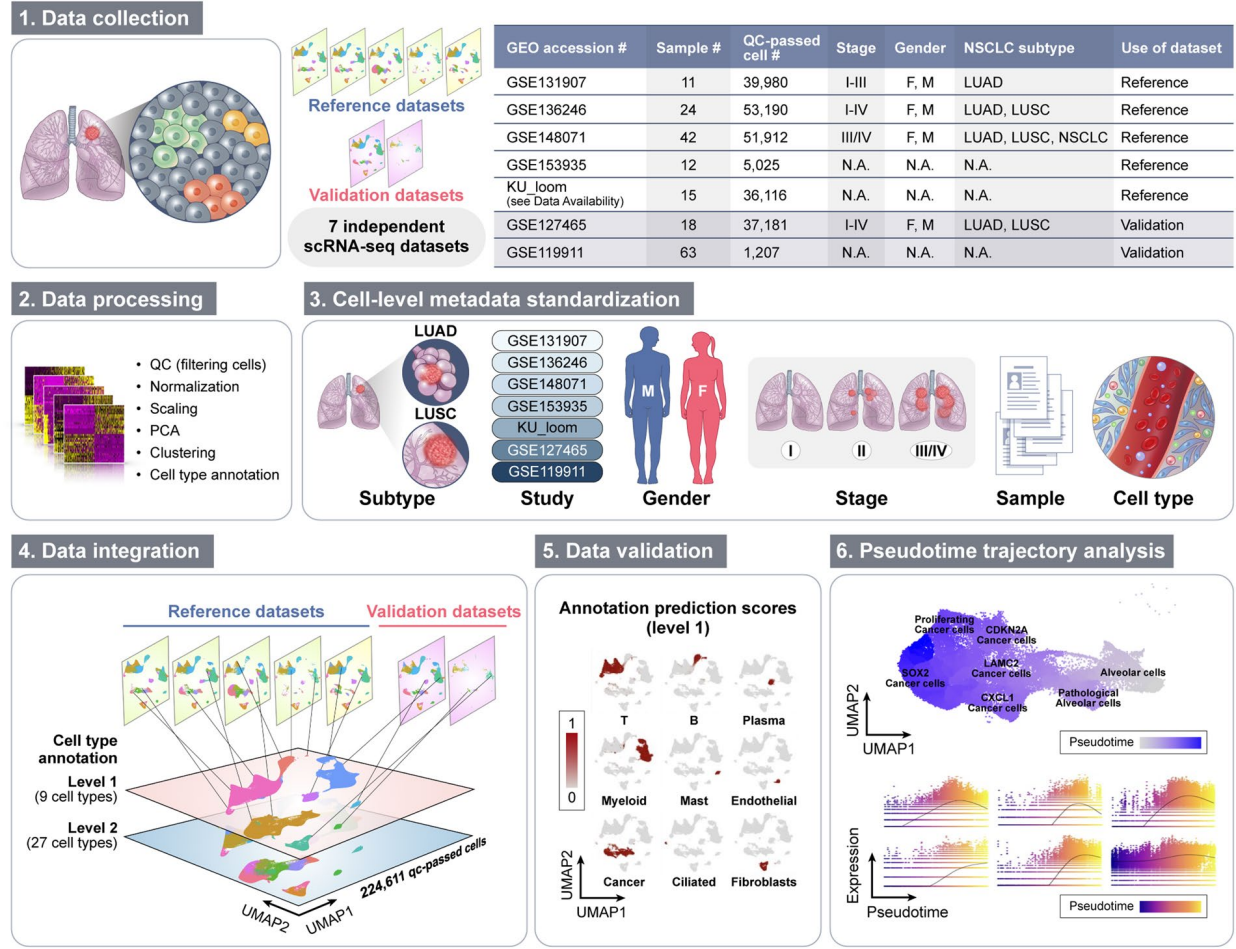
Study design. Seven independent datasets were collected, pre-processed, and clustered using the Seurat package. Cell-level metadata on cell type classification and sample clinical information was standardized for all datasets. To obtain a large reference dataset, five datasets were integrated in an anchor-based manner. The cells of the integrated reference were subjected to a standard workflow for clustering and cell type annotation. The integrated reference was used for annotation of the validation dataset and the two datasets were then merged into the final dataset. Additionally, pseudotime trajectory analysis of selected clusters was conducted.

Until now, published integrated lung datasets have been established for healthy tissue or single cell types^20,21^. However, a large-scale integrated data set of NSCLC, comprising data from several studies, and a variety of cell populations is still missing, up until very recently, there is a high-resolution single-cell atlas of the tumor microenvironment in NSCLC specifically^22^. Here, we present an integrated single-cell transcriptomic dataset for human NSCLC, containing 224,611 cells, with a thorough characterization of present cell types on two levels of annotation (Fig. 1). Our integrated transcriptome data may serve as a vast resource for studying gene expression patterns between cell types, reconstructing cellular trajectories and identification of potential novel biomarkers in NSCLC.

## Results

### Generation of an integrated reference dataset of NSCLC tumors

**Fig. 2:**
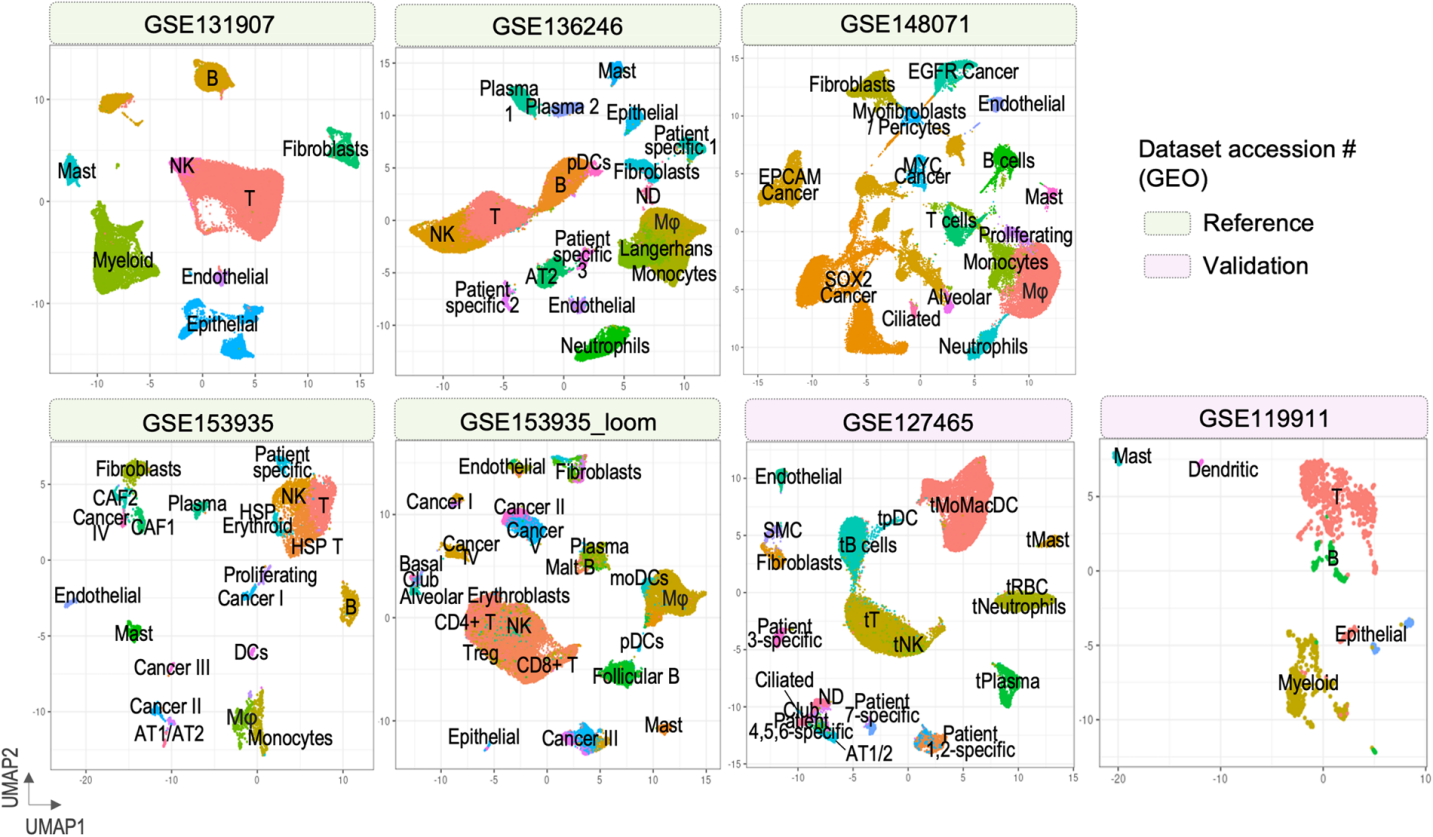
UMAP plots of the seven datasets after individual clustering analysis.

For generation of the large-scale integrated dataset, we collected seven publicly available scRNA-seq datasets comprising of 185 NSCLC human tumor samples in total. Among the seven datasets, five were used to construct an integrated reference and the remaining two served as validation. Details on samples included in the analysis are summarized in Tables 1,2. Using the R Seurat package (v 4.1.0)^8^ we followed a standard workflow for quality control and clustering of cells (Table 3). Each dataset was processed individually, including only human tumor samples. We identified diverse cell populations which were clearly separated on Uniform Manifold Approximation and Projection (UMAP) embeddings (Fig. 2).

**Table 1:** Naming and basic information on the datasets used in the study.

GEO accession #	Sample #	QC-passed cell #	Stage	Gender	NSCLC subtype	Use of dataset
GSE131907	11	39,980	I-III	F, M	LUAD	Reference
GSE136246	24	53,190	I-IV	F, M	LUAD, LUSC	Reference
GSE148071	42	51,912	III/IV	F, M	LUAD, LUSC, NSCLC	Reference
GSE153935	12	5,025	N.A.	N.A.	N.A.	Reference
Loom files (see Data Availability)	15	36,116	N.A.	N.A.	N.A.	Reference
GSE127465	18	37,181	I-IV	F, M	LUAD, LUSC	Validation
GSE119911	63	1,207	N.A.	N.A.	N.A.	Validation

**Table 2:** Accession numbers of the samples from each study used in the reanalysis.

Sample #	Dataset/sample accession #	Sample #	Dataset/sample accession #	Sample #	Dataset/sample accession #
	GSE131907	12	GSM4658775	15	GSM3387067
1	GSM3827125		**KU_loom**	16	GSM3387068
2	GSM3827126	1	1	17	GSM3387069
3	GSM3827127	2	2	18	GSM3387071
4	GSM3827128	3	3	19	GSM3387072
5	GSM3827129	4	4	20	GSM3387073
6	GSM3827130	5	5	21	GSM3387074
7	GSM3827131	6	6	22	GSM3387075
8	GSM3827132	7	8	23	GSM3387077
9	GSM3827133	8	9	24	GSM3387078
10	GSM3827134	9	10	25	GSM3387079
11	GSM3827135	10	12	26	GSM3387080
	**GSE136246**	11	13	27	GSM3387081
1	GSM4043237	12	14	28	GSM3387082
2	GSM4043238	13	16	29	GSM3387083
3	GSM4043239	14	17	30	GSM3387084
4	GSM4043240	15	18	31	GSM3387086
5	GSM4043241		**GSE127465**	32	GSM3387089
6	GSM4043242	1	GSM3635278	33	GSM3387090
7	GSM4043243	2	GSM3635279	34	GSM3387091
8	GSM4043244	3	GSM3635280	35	GSM3387092
9	GSM4043245	4	GSM3635281	36	GSM3387098
10	GSM4043246	5	GSM3635285	37	GSM3387099
11	GSM4043247	6	GSM3635286	38	GSM3387100
12	GSM4043248	7	GSM3635288	39	GSM3387101
13	GSM4043249	8	GSM3635289	40	GSM3387104
14	GSM4043250	9	GSM3635290	41	GSM3387105
15	GSM4043251	10	GSM3635292	42	GSM3387106
16	GSM4043252	11	GSM3635293	43	GSM3387107
17	GSM4043253	12	GSM3635294	44	GSM3387110
18	GSM4043254	13	GSM3635296	45	GSM3387112
19	GSM4043255	14	GSM3635297	46	GSM3387113
20	GSM4043256	15	GSM3635298	47	GSM3387114
21	GSM4043257	16	GSM3635299	48	GSM3387115
22	GSM4043258	17	GSM3635301	49	GSM3387116
23	GSM4043259	18	GSM3635302	50	GSM3387117
24	GSM4043260		**GSE119911**	51	GSM3387118
	**GSE148071**	1	GSM3387051	52	GSM3387121
1–42	GSM4453576–4453617	2	GSM3387052	53	GSM3387122
	**GSE153935**	3	GSM3387053	54	GSM3387123
1	GSM4658758	4	GSM3387054	55	GSM3387127
2	GSM4658760	5	GSM3387055	56	GSM3387128
3	GSM4658762	6	GSM3387056	57	GSM3387135
4	GSM4658763	7	GSM3387057	58	GSM3387138
5	GSM4658764	8	GSM3387058	59	GSM3387143
6	GSM4658765	9	GSM3387059	60	GSM3387146
7	GSM4658767	10	GSM3387060	61	GSM3387150
8	GSM4658768	11	GSM3387061	62	GSM3387153
9	GSM4658770	12	GSM3387062	63	GSM3387155
10	GSM4658772	13	GSM3387063		
11	GSM4658774	14	GSM3387064

**Table 3:** Analysis of the seven scRNA-seq datasets in R Seurat- workflow.

Dataset	GSE131907	GSE136246	GSE148071	GSE153935	KU_loom	GSE127465	GSE119911
Step 1: QC	nFeature_RNA > 200 & < 3000; Percent_mt <20						
	Step 2: Normalization						
	Step 3: Identification of variable features						
	Step 4: Scaling the data						
	Step 5: PCA dimensional reduction						
Step 6: Determine no. of PCs	20	20	20	20	20	20	20
	Step 7: Cell clustering						
	Step 8: UMAP plotting						
Step 9: Cell type annotation	Annotation provided	Annotation provided	Own annotation	Own annotation	Own annotation	Annotation provided	Own annotation

**Fig. 3:**
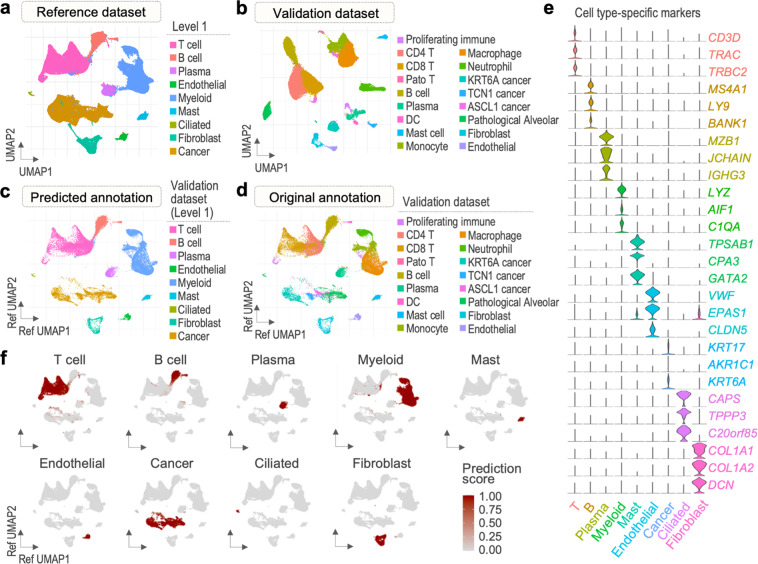
Transfer of reference cell type labels to cells of the validation dataset. (**a**) Cell types of the reference dataset (level 1). (**b**) Cell types of the validation dataset (original annotation). (**c**) Cells of the validation dataset projected in a UMAP structure of the reference with predicted annotation (level 1). (**d**) Cells of the validation dataset projected in a UMAP structure of the reference with original annotation. (**e**) Violin plots of reference cell type-specific markers expression in the validation cells (level 1). (**f**) Validation cell type prediction scores (level 1).

Subsequently, we integrated the five reference datasets using identified integration anchors and performed the downstream analysis. The reference dataset comprised of 186,223 cells, distributed among 27 clusters (Supplementary Fig. 1a–e). By examining expression patterns of canonical marker genes (see details in the “Methods” section), we performed a two-level classification of clusters, in which 9 and 27 cell types were identified for level 1 and 2 annotation, respectively (Fig. 3a, Supplementary Fig. 1f, g). The main cell types include immune (T, B, plasma, mast, and myeloid cells), epithelial (cancer and ciliated cells), and stromal cells (fibroblasts and endothelial cells), all of which were further divided into subtypes in level 2 annotation.

### Use of the reference dataset for annotation of query datasets

We integrated the two validation datasets via the anchor-based approach to obtain one validation dataset comprised of 39,511 cells. We clustered the cells of the validation dataset into 17 clusters, in which we initially classified independently of the reference dataset using canonical marker genes (Fig. 3b, Supplementary Fig. 2a–f). To assess the validity of the reference dataset, we conducted a cell type label transfer from the reference onto the validation dataset (Fig. 3c, Supplementary Fig. 2g). As a result, we obtained two levels of predicted annotations for the validation dataset. Cells of the validation dataset were well distributed in UMAP structure of the reference dataset, and all cell types defined in the reference were identified in the validation. We observed a satisfactory match between the original and predicted validation annotation in terms of main cell types, supporting the technical quality of our integrated data as an annotation reference atlas (Fig. 3d).

Next, we assessed the accuracy of the annotation predictions obtained in the mapping process. The cells of the validation dataset showed cell type-specific expression of marker genes (Fig. 3e) and high prediction score computed by the Seurat for all major cell types (Fig. 3f, Supplementary Fig. 3), supporting the credibility of the predicted annotations. To avoid inclusion of faultily classified validation cells in the final dataset, only the cells with high prediction score (>0.5) were merged into the final dataset and were selected as default identities of the validation dataset for further analyses.

### Cell type classification of the final dataset

**Fig. 4:**
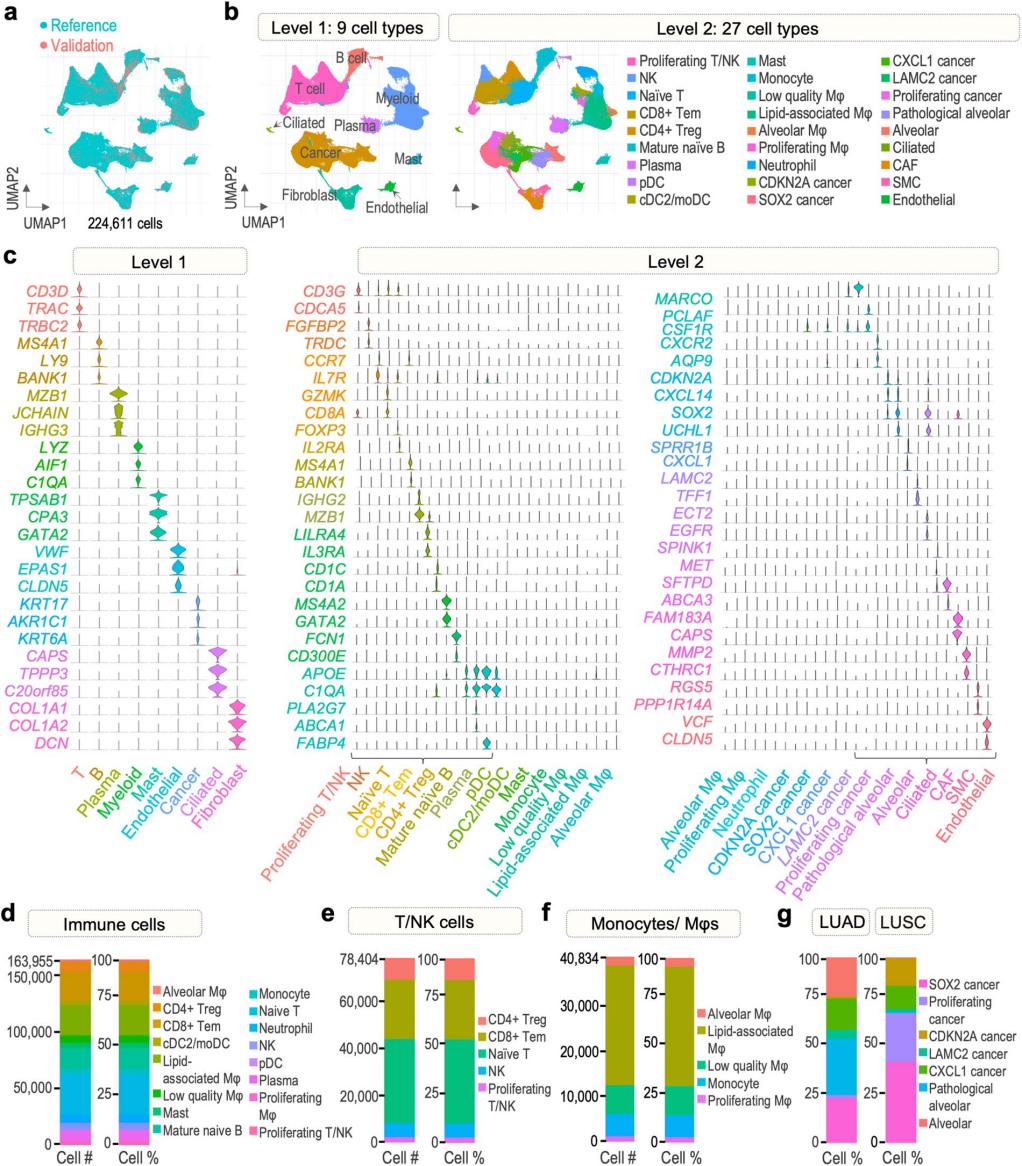
Cell types of the final dataset. (**a**) Distribution of cells derived from the reference and validation datasets in the final dataset. (**b**) Two levels of cluster annotation. (**c**) Violin plots of cell type-specific gene markers used for level 1 and 2 annotations. (**d**) Number of cells from immune cell population and their proportion. (**e**) Number of cells from population of T and NK cells and their proportion. (**f**) Number of cells from population of monocytes and macrophages and their proportion. (**g**) Proportion of cells from cancer cell population in LUAD and LUSC samples.

We merged the reference and validation datasets into a final dataset comprised of 224,611 cells. The UMAP plot in Fig. 4a shows a clear overlap of cells from the validation dataset with the reference in a single UMAP embedding, demonstrating a successful incorporation of the two datasets. We defined the previously generated two levelled annotation as final cell type classification of the final dataset (Fig. 4b,c).

We aimed at thoroughly characterizing the immune infiltrate and expression patterns of immune cells that reside in the tumor microenvironment (TME), including diverse subpopulations of T cells and myeloid cells (Fig. 4d–f, Supplementary Fig. 4). Subtyping of the T cell cluster revealed that naïve T cells accounted for majority of all T cells (45.41%), followed by CD8+ effector memory T cells (Tem), CD4+ regulatory T cells (Treg), NK, and proliferating T cells (32.29, 11.76, 7.68, and 2.88%, respectively). We found lipid-associated macrophages to be the most abundant subtype of the monocyte/macrophage group (64.70%). The remaining subtypes included low-quality macrophages, monocytes, alveolar, and proliferating macrophages (15.56, 12.01, 4.75, and 2.98%, respectively). Among other immune cells, we found a considerable amount of mature naïve B cells (11.24% of all immune cells), plasma cells (5.66%), and neutrophils (4.45%). Moreover, a detectable level of mast cells (2.85%) and dendritic cells (conventional/monocyte-derived 2.48%, plasmacytoid 0.59%) was identified. These results highlight the diversity of the immune cell population in the TME of NSCLC and provide a field of action for future studies.

We next identified seven subclusters in the cancer cluster, including alveolar cells, pathological alveolar cells and five cancer cell subtypes. We classified the cancer cells into the five cancer subtypes as CDKN2A, SOX2, CXCL1, LAMC2, and proliferating cancer based on the top markers that are highly expressed in each cluster. Interestingly, we found substantial differences in proportions of cancer cell subtypes between LUAD and LUSC samples (Fig. 4g). In LUAD, the proportion of alveolar (21.05% vs 0.5%), pathological alveolar (30.07% vs 0.34%) was much higher comparing to LUSC, in line with the previously reported LUAD developing from alveolar cells^16^. LUAD samples were also characterized by a higher percentage of LAMC2 (4.97% vs 1.79%) and CXCL1 cells (16.95% vs 12.5%). As CXCL1 and LAMC2 are associated with recruitment of neutrophils and macrophages into tumor tissue^23,24^, these results demonstrate the significant role of immune cell population in LUAD growth. In contrast to LUAD, LUSC samples were more abundant in CDKN2A (14.65% vs 1.05%), proliferating (26.33% vs 1.73%), and SOX2 cancer cells (43.89% vs 24.18%). Tumor suppressor CDKN2A regulates the cell cycle and is frequently altered in LUSC^25^. Similarly, SOX2 controls cell proliferation and is commonly amplified in LUSC, promoting its growth by maintaining stem cell-like phenotype of cancer cells^26^. Together, these three cell subtypes account for over 80% of all cancer cells derived from LUSC samples, indicating the highly malignant nature of this tumor subtype.

### Assessment of the validity of the final integrated dataset

**Fig. 5:**
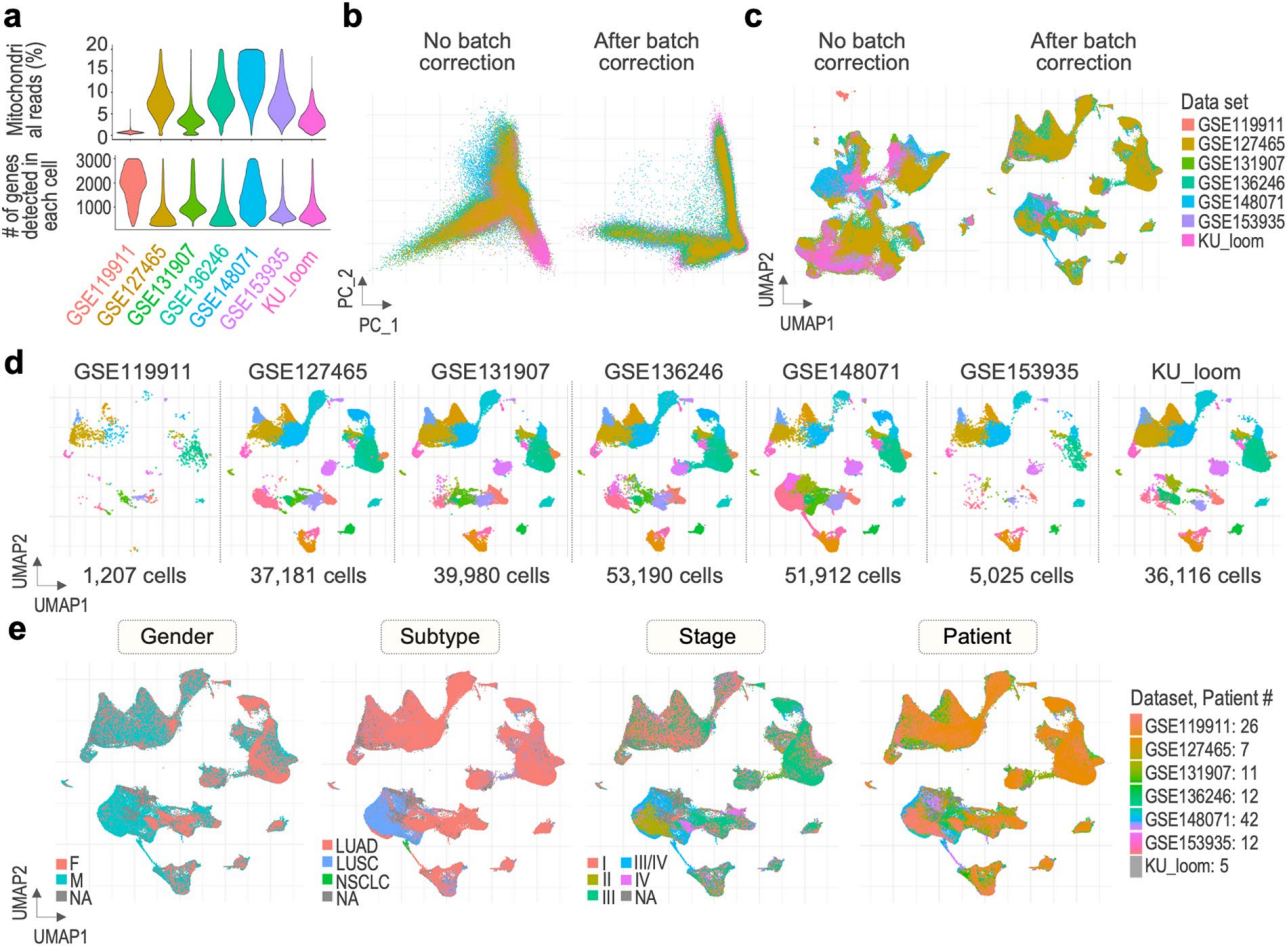
Quality of the final dataset. (**a**) Violin plots showing QC metrics of cells included in the final dataset, including percentage of counts from mitochondrial genes (Percent_mt) and number of genes (nFeature_RNA), split by study. (**b**) Removal of the batch effect presented by PCA and dimensional reduction plots (**c**) for the final dataset, in a comparison to a merged dataset without batch correction. (**d**) Distribution of cells from the seven analyzed datasets in the clusters of the final dataset. (**e**) UMAP plots of the final dataset cells grouped by clinical metadata, including gender, subtype, stage, and patient.

For quality control of our final dataset, we applied commonly used quality metrics such as percentage of counts from mitochondrial genes and number of features (Fig. 5a). Cells that have more than 20% of mitochondria-related read counts or unique feature counts over 3,000 and less than 200 were filtered out. To visualize the efficiency of the integration process, we generated PC and dimensional reduction plots comparing our final dataset and a dataset comprised of the same datasets, merged without batch correction. The resulting plots in Fig. 5b show a major disconnection between the merged data when colored by dataset in the first two PCs. In contrast, the final data clearly overlays between the source datasets, suggesting that the effect of non-biological variances have been corrected. Cells of the batch-uncorrected dataset are separated by study of origin, rather than cell type, whereas those of the batch-corrected final dataset are distributed more evenly according to study in every cluster (Fig. 5c), suggesting cells are grouped by cell type that account for the most variance in the data. The distribution of cells in the UMAP plot visualized in Fig. 5d once again shows that cells from each study can be found in each cluster, suggesting that the differences in contribution to formation of the clusters arise from the count of cells in the initial data sets, rather than differences in cell type composition. Altogether, these results indicate that the process of integration and data transfer with Seurat was completed successfully, minimizing the effect of technical batches on cell clustering. An additional value of our dataset is the collected metadata containing clinical information on patients included in the study, such as gender, histological subtype, and stage of the tumor (Fig. 5e).

### Pseudotime trajectory analysis

**Fig. 6:**
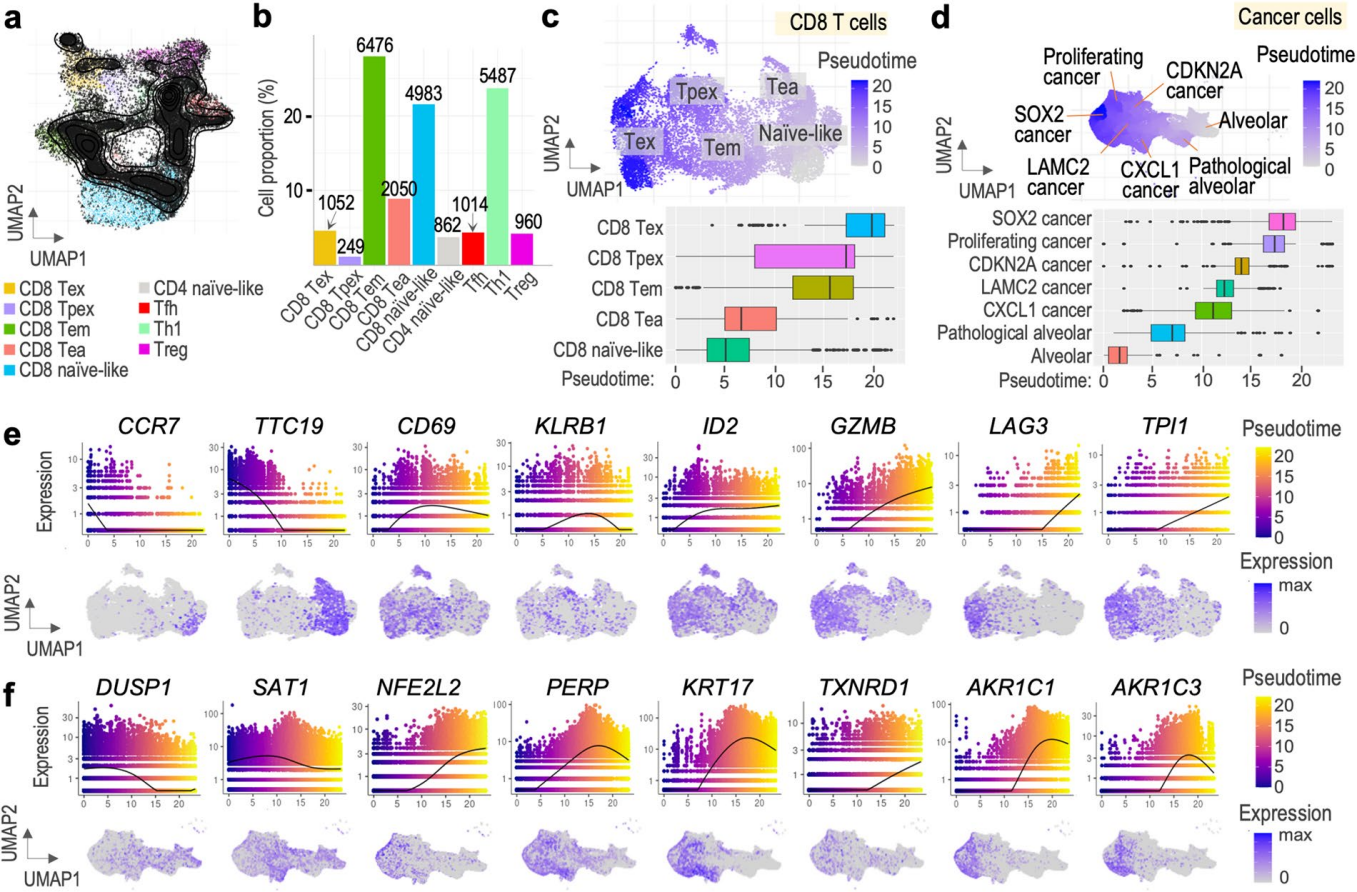
Pseudotime trajectory analysis. (**a**) Distribution of query T cells on ProjecTILs reference map. (**b**) Percentage and number of T cells from each functional state. (**c**) UMAP plot of CD8+ T cell subset colored by pseudotime (top) and boxplot showing median pseudotime of each cell type (bottom). (**d**) UMAP plot of cancer cell subset colored by pseudotime (top) and boxplot showing median pseudotime of each cell type (bottom). (**e**) Chosen genes of the CD8+ T cell subset showing changing expression in pseudotime. (**f**) Chosen genes of the cancer cell subset showing changing expression in pseudotime.

T cells are the main target of immunotherapy in NSCLC^27,28^. According to current understanding of CD8+ T cell differentiation, upon activation naïve T cells differentiate into different effector and memory T cells. In tumors, chronic T cell stimulation leads to disturbance in their differentiation toward dysfunction and exhaustion characterized by loss of effector function and expression of inhibitory receptors^29^. To depict the different states of CD8+ T cells, we conducted a pseudotime trajectory analysis using the R Monocle3 package^7^. Specifically, we extracted T cells from our final dataset and reanalyzed their cell states using R ProjecTILs package^30^. We projected our query cells on the reference map provided by ProjecTILs and calculated the number of cells in each state (Fig. 6a,b, Supplementary Fig. 5). In total, 14,810 cells were classified as ‘CD8_NaiveLike’, ‘CD8_EarlyActiv’, ‘CD8_EffectorMemory’, ‘CD8_Tpex’, or ‘CD8_Tex’ cells for subsequent analyses. The extracted cells were re-clustered using Seurat and subjected to trajectory analysis via Monocle3. As T cells differentiate from naïve to effector to memory and exhausted states, we specified the trajectory to start from CD8_NaiveLike cells. The UMAP plot in Fig. 6c shows the population of CD8+ T cells colored by pseudotime, suggesting a continuous progression of cells from naïve-like to exhausted state. Ordering the five cell states by median pseudotime revealed a transition from naïve-like cells to early activated, followed by effector memory, precursor exhausted, and exhausted cells (Fig. 6c, bottom). Importantly, although the median pseudotime of Tpex cluster is higher than that of Tem, it exhibits a wider spectrum of pseudotime values, suggesting that initiation of T cell exhaustion may start upon activation. We further verified these results by analysing genes which showed significant expression changes in pseudotime. We observed clear differences in expression of naïve (CCR7, TTC19), memory (CD69, ID2), cytotoxicity (KLRB1, GZMB), and exhaustion-related genes (LAG3, TPI1) in pseudotime, supporting a consistent shift of T cells towards differentiation and exhaustion (Fig. 6f).

Lastly, we performed a joint trajectory analysis of all 46,450 cells of the cancer cluster. Starting from alveolar cells, the cells transformed into pathological alveolar cells, CXCL1, LAMC2, CDKN2A, proliferating, and SOX2 cancer cells as they progressed in pseudotime (Fig. 6d). We identified distinct changes in expression of reactive oxygen species (ROS) genes in pseudotime (Fig. 6f). Expression of DUSP1 was the highest at the beginning of pseudotime, as opposed to TXNRD1 which was mainly expressed in late pseudotime. It has been suggested that high expression of DUSP1 is correlated with better prognosis, whereas TXNRD1, with poor patient prognosis in lung cancer^31^. These results demonstrate progression of cancer cells in the trajectory towards more resistant phenotype. Moreover, few genes have been reported to be implicated in p53 signalling (SAT1, PERP, KRT17)^32–34^ or ferroptosis (SAT1, NFE2L2, AKR1C1, AKR1C3)^32,35,36^. Together, the presented dataset reveals complete cancer cell landscape of NSCLC tumor progression, associated with ROS metabolism and p53 activity.

## Discussion

In this study, we generated a large-scale scRNA-seq dataset of human primary NSCLC tumors containing both LUAD and LUSC samples, from early to advanced stages, of both genders. While each dataset used to generate the presented data contains a limited number of tumor cells, our integrated dataset may provide a more comprehensive cell landscape of NSCLC specifically. We thoroughly annotated the presented scRNA-seq data to facilitate the re-use of our data for novel cell type discovery and extensive characterization of diverse cell subpopulations, including immune cells residing in tumor microenvironment. In addition, inclusion of patients from different studies with standardized cell-level metadata may enable the study of NSCLC transcriptome on a wider spectrum of samples than the analysis of single study or dataset having limited number of QC-passed cells.

Since in this analysis we reused data from published studies, we observed substantial batch effects arising from the technical differences in library preparation and data processing. According to several benchmarking studies evaluating performance of available batch effect correction methods, Harmony and Seurat are described as tools suitable for scRNA-seq analysis. As Harmony utilizes PCA subspace as input for further transformations, it is often noted to be faster and require less memory. However, it limits its usability in gene-based analyses in which expression matrix is the input, such as pseudotime or identification of differentially expressed genes^9,10^. Integration with Seurat usually requires more memory and a longer runtime. Nevertheless, it can precisely merge batches while producing a corrected gene expression matrix, useful for downstream analysis^9,10^. Seurat also enables data transfer between scRNA-seq datasets. In data transfer, PCA structure of a reference dataset is projected onto the query based on transfer anchors, and annotation predictions are generated for query cells. This workflow does not require CCA, which substantially reduces the runtime^11^. Taken together, although Harmony may be faster in the process of integration itself, we employed functions of the Seurat, which allows a wider range of downstream analyses, to integrate the datasets and correct for batch effects. In addition, we used the same pre-processing and clustering workflow on each dataset prior to integration, to minimize potential differences between them. We used PCA and UMAP to visualize batch effect correction, which showed good batch mixing results in our final dataset in comparison to a dataset obtained using basic merging function.

Through extensive analysis of marker genes’ expression, we classified the 224,611 cells into nine main cell types, which were further divided into twenty-seven subtypes primarily consisting of immune cell populations. Apart from cell types commonly described in NSCLC microenvironment, we identified a subtype of low-quality macrophages characterized by elevated expression of mitochondrial genes and genes encoding for ribosomal proteins, suggesting damage or stress of the cells. We found that the most abundant subtype of macrophages show a lipid-related signature, with expression of PLA2G7, ABCA1, FOLR2, APOE, CTSB/D, and C1QA/B/C, which is associated with phagocytosis and immunosuppression^37^. Sub-clustering of T cells further revealed the presence of naïve, helper and cytotoxic cells, as well as NK and proliferating T cells. Comparing cell type abundances between our dataset and the recently published NSCLC atlas (Salcher *et al*.)^22^, we observed several differences in fractions of cell types. Interestingly, neutrophils, which are short-lived cells, often underrepresented in scRNA-seq studies, in our dataset account for 3.25% of all cell populations, while in the Salcher *et al*. dataset^22^, only 1.5%. In addition, fractions of epithelial cells and B cells are higher in our dataset (20.99% vs ~15% and 8.64% vs ~5.5%, respectively). In contrast, abundance of macrophages/monocytes is lower in our dataset than in the Salcher *et al*. dataset (18.18% vs 28,5%). Nevertheless, we identified several subtypes of myeloid cells showing distinct signatures, as noted above.

We conducted an additional functional state analysis of the T cells using ProjecTILs^30^ and subjected a subset of CD8+ cells to pseudotime trajectory analysis via Monocle3^7^. The mouse-derived reference map provide by ProjecTILs may attribute to a large number of our query cells that were filtered out during QC process. Species-specific differences in gene expression may have contributed to failure in detecting the query cells as “pure” T cells. However, we believe that the remaining QC-passed 14,810 cells which were successfully assigned to reference functional states were sufficient to perform a trajectory analysis. Our analyses revealed a dynamic functional spectrum of CD8+ T cells from naïve to exhausted state in NSCLC, showing effective data reuse.

Finally, we identified seven cancer subclusters and analyzed possible dynamics between them in pseudotime. The seven subclusters included alveolar cells, pathological alveolar cells expressing both normal respiratory cell markers (SFTPB, AGR3) and genes related to cancer progression (SPINK1, MET), as well as five cancer subsets. We observed considerable differences in abundance of cells from each of the seven subtypes between LUAD and LUSC samples, implicating stem cell-like phenotype of LUSC cells and immune infiltration promotion by LUAD. Pseudotime trajectory analysis revealed a dynamic path in which normal epithelial cells went under a transformation to cancer cells. This process was accompanied by changes in expression of genes related to p53 signaling and ROS metabolism, showing further differences in progression of the two tumor subtypes. Interestingly, several genes (PERP, KRT17, AKR1C1) have been recently reported as potential NSCLC biomarkers^36,38^. As we previously noted, LUAD and LUSC showed distinct differences in cancer cell subtype content. The cell types more abundant in LUSC (SOX2, CDKN2A, proliferating cancer) were placed later in pseudotime than the LUAD-specific cell types (alveolar, pathological alveolar, CXCL1 cancer). Altogether, these results suggest that LUSC cells show more aggressive and resistant characteristics. In conclusion, these results demonstrate the usefulness and technical validity of our integrated scRNA-seq dataset. Reuse of this large-scale dataset may contribute to further understanding of NSCLC.

## Methods

### Data collection and pre-processing

Seven publicly available scRNA-seq datasets were collected, comprising of 185 NSCLC human primary tumor samples in total. Datasets GSE131907^28,39^, GSE136246^40,41^, GSE148071^42,43^, GSE153935^44,45^, and KU_loom (https://gbiomed.kuleuven.be/scRNAseq-NSCLC)^46,47^ were used to create a large reference dataset, whereas datasets GSE127465^27,48^ and GSE119911^49,50^ served as validation. Details on samples included in the analysis are summarized in Tables 1, 2. Using Seurat package (v 4.1.0)^8^ in R (v 4.1.1), a standard workflow for data pre-processing and the clustering of cells was followed. Briefly, the seven scRNA-seq datasets were analyzed individually, including quality control (QC), normalization, feature selection, data scaling, dimensional reduction by principal component analysis (PCA), clustering, Uniform Manifold Approximation and Projection (UMAP) reduction, and visualization of clusters. From each dataset, human tumor samples were extracted and loaded into respective Seurat objects. QC of the gene-cell matrix consisted of filtering the cells such that cell with counts from mitochondrial genes below 20 percent and number of features more than 200 and less than 3000 were included. Detailed information on quality control and subsequent steps of the single data sets analysis are described in Table 3. Gene expression normalization was applied to each dataset using LogNormalize method. The number of principal components (PCs) to include in further analysis was determined based on JackStraw plots and Elbow plots generated for each dataset. Cell clustering was conducted using FindNeighbors and FindClusters functions, and non-linear dimensional reduction was managed by RunUMAP function. For datasets GSE131907^28,39^, KU_loom (https://gbiomed.kuleuven.be/scRNAseq-NSCLC)^46,47^, and GSE127465^27,48^, metadata on cell type annotation of the single cells was provided by the authors. Clusters from the remaining datasets were assigned to specific cell types considering positive (avglog2FC > 0) cell type-specific markers found via FindAllMarkers function. For visualization, UMAP plots showing obtained annotated clusters were generated (Fig. 2).

### Integration of reference datasets

To establish a single reference dataset, five datasets (GSE131907^28,39^, GSE136246^40,41^, GSE148071^42,43^, GSE153935^44,45^, and KU_loom (https://gbiomed.kuleuven.be/scRNAseq-NSCLC)^46,47^) were integrated and analyzed using functions of the Seurat package, following the workflow proposed by Satija Lab^11,12^ (https://satijalab.org/seurat/articles/integration-introduction.html). A list consisting of five pre-processed datasets previously specified as reference was created and features repeatedly shared within the objects were identified using Seurat’s SelectIntegrationFeatures function. Subsequently, FindIntegrationAnchors function enabled selection of a set of 219,432 cell pairs in a similar biological state (anchors), which were then utilized in the integration process via IntegrateData function. Once the integration process was executed successfully, the integrated assay was specified as default for downstream analysis.

### Reference dataset analysis

The integrated dataset comprised of 186223 cells. Standard steps leading to clustering of the cells were conducted, including identification of highly variable features, scaling of the data, PCA, UMAP (no. of dims = 30), and finding neighbours (Supplementary Fig. 1). Identification of clusters was performed at resolutions 0.02 and 0.5 respectively, to obtain two versions of dimension reduction plots containing different number of clusters (level1 and level2). The clusters were classified using two types of gene markers: positive biomarkers detected using FindAllMarkers function, and markers conserved across the datasets detected via FindConservedMarkers function (grouping.var = Study, DefaultAssay = RNA). The cell type identities were firstly assigned to clusters based on the conserved markers, while the general biomarkers were a secondary source of information for both levels of annotation. Since identification of conserved markers is based on differential expression testing, the RNA assay was used in this analysis instead of the integrated assay, to include more potential markers. Features conserved among the data sets were identified using study of origin as the grouping variable. Cell type specificity of the markers was further confirmed using several recent publications^28,37,51–78^. As a result, 9 and 27 cell types were found for level 1 and 2 of annotation, respectively (Supplementary Fig. 1, Table 4).

**Table 4:** Additional marker genes used for cell type classification of clusters (level 2).

Level2 annotation – additional markers	
Proliferating T/NK	TOP2A, MKI67, NUSAP1
NK	KLRF1, KLRD1, KLRB1, GNLY, NKG7
Naïve T	PTPRC
CD8+ Tem	GZMA, GZMM, CD8
CD4+ Treg	CTLA4, CD4
Mature naïve B	CD22, CD53, CD79A
Plasma	IGHA2, IGHM, TNFRSF17
pDCs	IRF7, IRF8
cDC2/moDCs	CLEC10A
Mast	KIT, CPA3, CD63
Monocytes	CD14, CSF3R
Low quality Mφ	LYZ, FTL, high number of MT- and RPL/S genes
Lipid-associated Mφ	MS4A7, IL1B, IL4I1, FOLR2, APOE, C1QA/B/C, CTSB/D
Alveolar Mφ	MCEMP1, PPARG, MRC1
Proliferating Mφ	CDCA8, MKI67, CENPF, CD14, TOP2A
Neutrophils	FCGR3B, CSF3R, S100A12, S100A8
CDKN2A Cancer	CDK4, PUM3, NTS, EPCAM
SOX2 Cancer	KRT17, S100A2, SFN, PTHLH, PERP
CXCL1 Cancer	SPRR3, AGR2, CEACAM6
LAMC2 Cancer	FGB, FGA, FGG, PAEP, TESC
Proliferating Cancer	MKI67, TOP2A, CENPF, CDC20
Pathological Alveolar	SFTPB, WFDC2, AGR2, AGR3, MUC1
Alveolar	SFTPC, AQP4, SCGB3A1
Ciliated	FOXJ1, CDHR3
CAF	SPARC, FAP, PDGFRB
SMC	ACTA2, CALD1, TAGLN
Endothelial	FLT1, PECAM1

### Validation dataset analysis

Datasets GSE127465^27,48^ and GSE119911^49,50^ acquired from NCBI GEO were processed individually with the previously described workflow for clustering analysis (Table 3). For dataset GSE127465^27,48^ identified clusters were annotated based on metadata provided by the authors, whereas clusters of dataset GSE119911^49,50^ were annotated manually based on canonical markers (Fig. 2). Due to a small number of QC-passed cells from dataset GSE119911^49,50^ (1359 cells), Seurat anchor-based integration of cells from the two validation datasets was conducted to form a single validation dataset (see “Integration of reference datasets”). The integrated validation dataset included 39511 cells and was subjected to clustering analysis (no. of dims = 30). At resolution 0.5, 17 clusters were obtained and initially classified according to expression of conserved gene markers (see “Reference dataset analysis”, Supplementary Fig. 2a–f).

### Cell type label transfer from reference to validation dataset

Following Seurat anchor-based methodology for data transfer^11^ (https://satijalab.org/seurat/articles/multimodal-reference-mapping.html), cell type classifications of the integrated dataset were transferred onto the validation dataset. Cells of our reference dataset were utilized as reference and validation dataset, as query. Transfer anchors were identified using FindTransferAnchors function, with LogNormalize as normalization method, PCA as reduction, and number of dimensions 30. The anchorset was applied in the label transfer using MapQuery function, leading to creation of two levels of predicted annotations (Fig. 3, Supplementary Fig. 2g). To assess efficiency of the new query annotations, prediction scores were generated for each of the query cells. Cells with high prediction score (predicted.celltype.score >0.5) were included in further analysis. The integrated and validation datasets were merged into a final dataset comprising 224611 cells and visualized in UMAP embedding of the reference. Additionally, feature plots showing strength of the cell type predictions were generated (Fig. 3f, Supplementary Fig. 3). The two levelled annotation was used as final classification of cells of the final dataset. Expression of marker genes and proportions of cell types were investigated (Fig. 4, Supplementary Fig. 4).

### Visualization of batch effect correction and final dataset quality

Violin plots showing QC metrics applied during pre-processing of the seven datasets were generated, including percentage of mitochondrial reads and number of genes detected in each cell (Fig. 5a). To assess the efficiency of the integration process, several visualization methods were used to compare our final dataset with a simply merged dataset without batch effect correction. A list of the seven pre-processed datasets was created and all respective Seurat objects were merged using Merge_Seurat_List function. The merged dataset was subjected to clustering analysis in a way corresponding to clustering of the reference and validation datasets (identification of highly variable features, scaling of data, PCA, UMAP (30 dims), finding neighbours, identification of clusters at resolution 0.5). PCA and dimensional reduction plots were visualized for both the final and merged datasets (Fig. 5b,c). UMAP plot of the cells of the final dataset split by study of origin was made to observe the placement of cells from each dataset (Fig. 5d). In addition, plots of the final UMAP structure colored by collected metadata were generated, including gender, histological subtype, stage of the tumor, and patient id (Fig. 5e).

### Pseudotime trajectory analysis

#### CD8+ T cells

Cells of the T cell cluster according to level 1 of annotation were extracted from the final dataset into a new Seurat object. Cell states of the T cells were re-evaluated using ProjecTILs R package (v 3.0)^30^. Reference atlas of tumor-infiltrating T lymphocytes was loaded from ProjecTILs Git repository. Our query T cells were filtered and projected on the reference map (Fig. 6a). Cell states predictions were generated according to gene expression signatures pre-determined by the package for specific T cell subtypes (Fig. 6b, Supplementary Fig. 5). Cells predicted as belonging to CD8+ T cell functional clusters were selected for further analysis, including CD8_NaiveLike, CD8_EarlyActiv, CD8_EffectorMemory, CD8_Tpex, and CD8_Tex. The newly obtained subset of cells was pre-processed using Seurat functions (FindVariableFeatures, ScaleData, RunPCA, FindNeighbors (dims = 1:20), FindClusters (resolution = 0.5), RunUMAP) and visualized using the annotations predicted by ProjecTILs. Pre-processed Seurat object was converted to an object of cell dataset class using as.cell_data_set function and data size factors were calculated using estimate_size_factors for trajectory analysis in Monocle3^7^ (v 1.0.0). Cell and gene-level metadata, counts and cluster information, as well as previously obtained UMAP embedding were retrieved from the Seurat object to the cell dataset object. All cells were assigned to a single partition and the trajectory graph was learned using learn_graph function. To place the cells in pseudotime, cells which belong to CD8_NaiveLike cluster were assigned as “roots” of the trajectory. Obtained cell pseudotime information was stored in the T cell Seurat object’s metadata for visualization purposes (Fig. 6c). Differential expression analysis was performed to identify genes of which expression changes in pseudotime (Fig. 6e). The top genes were found by arranging the results by q_value and status (status == “OK”).

#### Cancer cells

Cells belonging to clusters “Alveolar”, “CDKN2A Cancer”, “CXCL1 Cancer”, “LAMC2 Cancer”, “Pathological Alveolar”, “Proliferating Cancer”, and “SOX2 Cancer” in level 2 of annotation were extracted from the final dataset into a new Seurat object. The Seurat object containing cancer cells was converted to an object of cell dataset class. Size factors for each cell were estimated using estimate_size_factors function. Necessary metadata etc. was retrieved from the Seurat object as described above for the T cell analysis. The trajectory graph was learned using learn_graph function and cells belonging to the Alveolar cluster were assigned as “roots” of the trajectory for pseudotime analysis. Obtained cell level pseudotime information was stored in the cancer cell Seurat object’s metadata (Fig. 6d). Accordingly, differential expression analysis was performed to identify genes with changing expression in pseudotime, and the top genes were found by arranging the results by q_value and status (status == “OK”) (Fig. 6f).

## Supplementary information

Supplementary Figures

## Data Availability

Among input data processed in the reanalysis, six datasets were acquired from NCBI GEO (GSE131907^28,39^ (2020), GSE136246^40,41^ (2021), GSE148071^42,43^ (2021), GSE153935^44,45^ (2020), GSE127465^27,48^ (2019), GSE119911^49,50^ (2022)). Dataset referred to as KU_loom was downloaded from resources of the Ku Leuven Laboratory for Functional Epigenetics as “all cells” loom file (https://gbiomed.kuleuven.be/scRNAseq-NSCLC (2018)^46,47^). Set of samples used in this study is summarized Table 2. Seurat object of our final scRNA-seq dataset with UMAP embeddings can be found at figshare (10.6084/m9.figshare.c.6222221.v3)^79^. Associated data, including matrix of raw and normalized counts, and metadata (two levels of cell type annotation, validation dataset prediction scores, QC metrics, patient id, gender, study of origin, tumor subtype and stage) are available under the same figshare project as “RNA_rawcounts_matrix”, “Integrated_normalized_counts”, and “Metadata” files, respectively.

## Code availability

The main computational tools used in this study are R language based. Seurat^8^ was used for data pre-processing, integration, and label transfer between reference and validation datasets. ProjecTILs^30^ was used for interpretation of T cell states, and Monocle3^7^ was used for pseudotime trajectory analysis. The R codes used for pre-processing of the used datasets, reference and validation datasets analysis, and pseudotime trajectory analysis can be found at figshare as “NSCLC_data_reanalysis_codes” file (10.6084/m9.figshare.c.6222221.v3)^79^.
