# Supplementary material for: An integrated single-cell transcriptomic dataset for non-small cell lung cancer
Source: Sci Data. 2023 Mar 27;10:167. doi: 10.1038/s41597-023-02074-6 (PMC10042991; doi:10.1038/s41597-023-02074-6)
Supplement: Supplementary file 1 — Supplementary Figures [file 41597_2023_2074_MOESM1_ESM.pdf]

Supplementary Figures

**Description**	**Slide**
Supplementary Figure 1. Pre-processing and cell type annotation of the integrated reference data set.	2
Supplementary Figure 2. Pre-processing and cell type annotation of the integrated validation data set.	3
Supplementary Figure 3. Validation cell type prediction scores (level 2).	4
Supplementary Figure 4. Number and proportion of all cells from level 1 and 2 annotations.	5
Supplementary Figure 5. Radar plots showing gene expression signature of query cells and reference cells of the five CD8+ T cell subtypes.	6

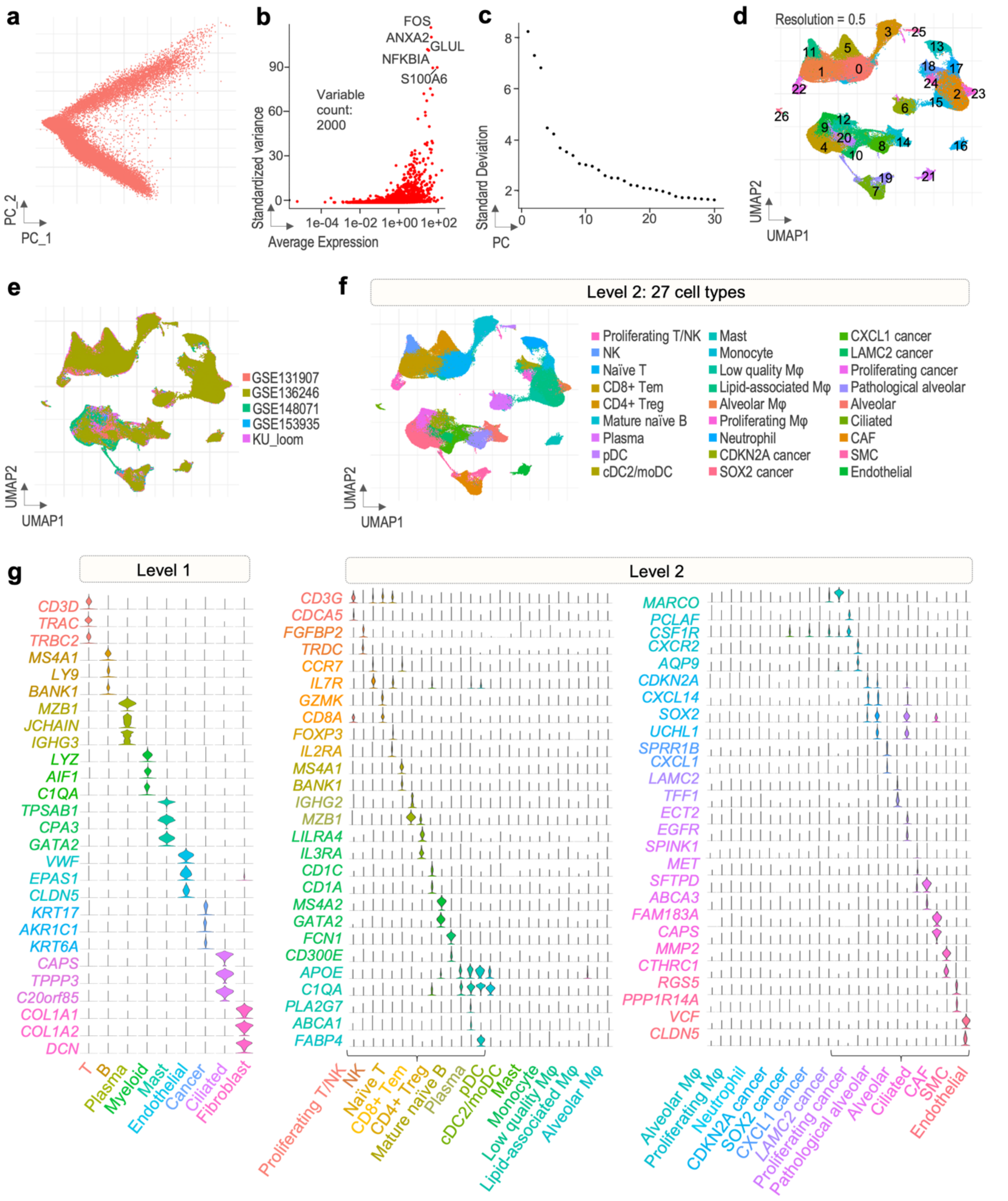

Supplementary Figure 1. Pre-processing and cell type annotation of the integrated reference data set. (a) PCA plot of the reference dataset. (b) Top 5 variable features in the reference data set. (c) Elbow plot showing a ranking by standard deviation for top 20 PCs. (d) Seurat clusters obtained for the reference data set. (e) Distribution of cells derived from the five original datasets in the reference dataset. (f) Cell types of the reference dataset (level 2). (g) Violin plots of cell type-specific gene markers used for level 1 and 2 annotations of the reference dataset.

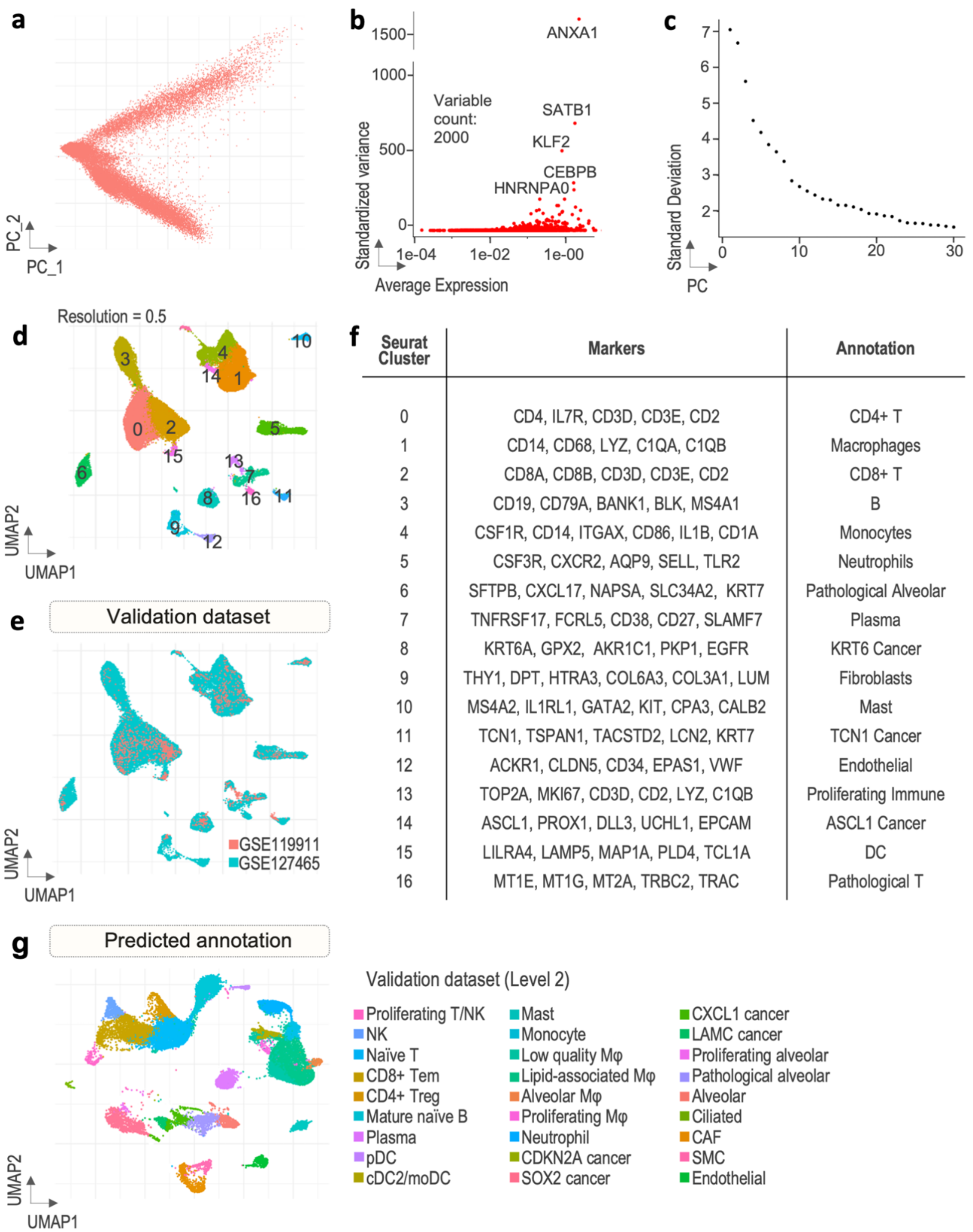

f

Seurat Cluster	Markers	Annotation
0	CD4, IL7R, CD3D, CD3E, CD2	CD4+ T
1	CD14, CD68, LYZ, C1QA, C1QB	Macrophages
2	CD8A, CD8B, CD3D, CD3E, CD2	CD8+ T
3	CD19, CD79A, BANK1, BLK, MS4A1	B
4	CSF1R, CD14, ITGAX, CD86, IL1B, CD1A	Monocytes
5	CSF3R, CXCR2, AQP9, SELL, TLR2	Neutrophils
6	SFTPB, CXCL17, NAPSA, SLC34A2, KRT7	Pathological Alveolar
7	TNFRSF17, FCRL5, CD38, CD27, SLAMF7	Plasma
8	KRT6A, GPX2, AKR1C1, PKP1, EGFR	KRT6 Cancer
9	THY1, DPT, HTRA3, COL6A3, COL3A1, LUM	Fibroblasts
10	MS4A2, IL1RL1, GATA2, KIT, CPA3, CALB2	Mast
11	TCN1, TSPAN1, TACSTD2, LCN2, KRT7	TCN1 Cancer
12	ACKR1, CLDN5, CD34, EPAS1, VWF	Endothelial
13	TOP2A, MKI67, CD3D, CD2, LYZ, C1QB	Proliferating Immune
14	ASCL1, PROX1, DLL3, UCHL1, EPCAM	ASCL1 Cancer
15	LILRA4, LAMP5, MAP1A, PLD4, TCL1A	DC
16	MT1E, MT1G, MT2A, TRBC2, TRAC	Pathological T

Supplementary Figure 2. Pre-processing and cell type annotation of the integrated validation data set. (a) PCA plot of the validation dataset. (b) Top 5 variable features in the validation data set. (c) Elbow plot showing a ranking by standard deviation for top 20 PCs. (d) Seurat clusters obtained for the validation data set. (e) Distribution of cells derived from the two original datasets in the validation dataset. (f) Table of cell type-specific gene markers used for

annotation of the validation dataset. (g) Cells of the validation dataset projected in a UMAP structure of the reference with predicted annotation (level 2).

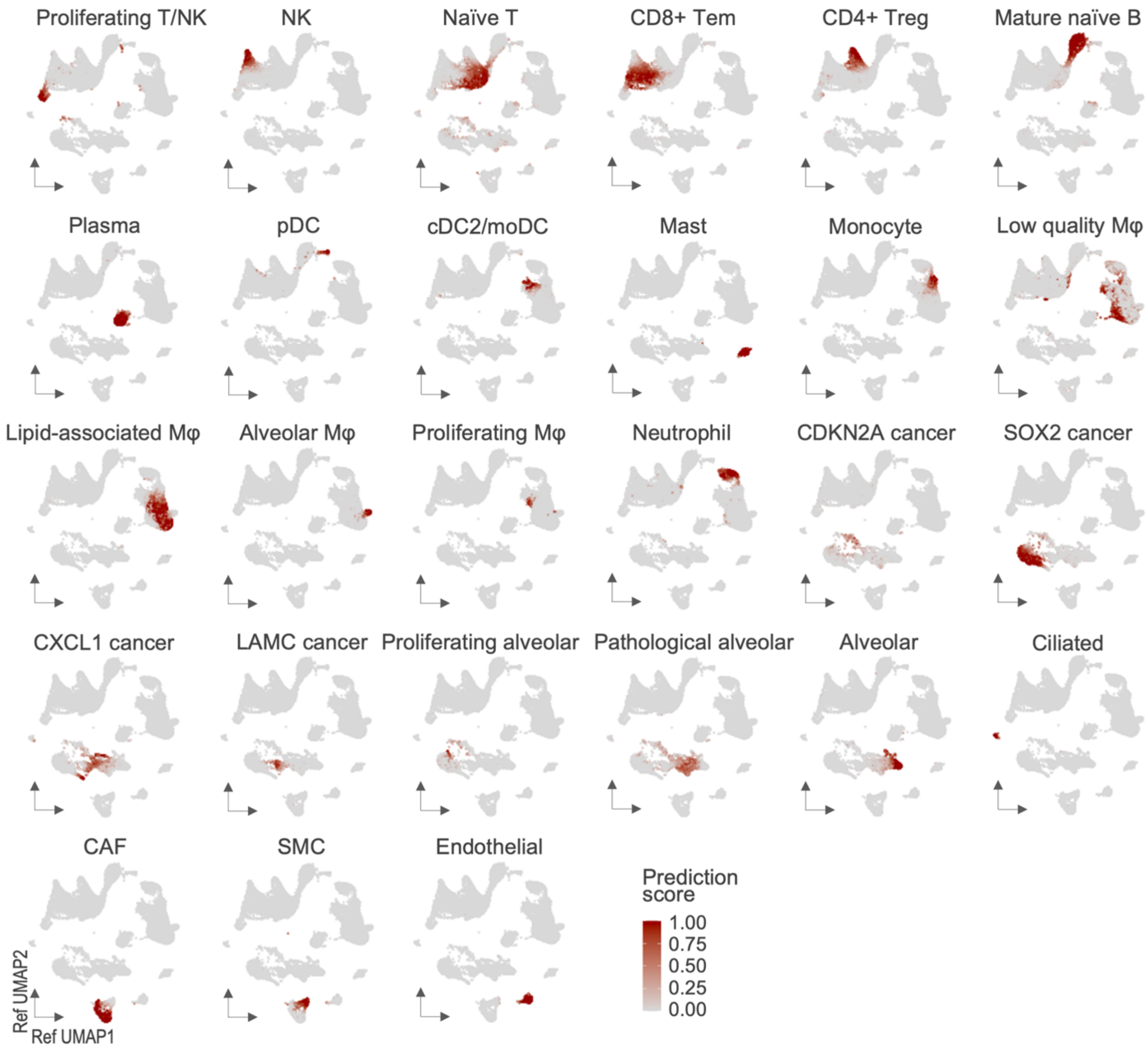

Supplementary Figure 3. Validation cell type prediction scores (level 2).

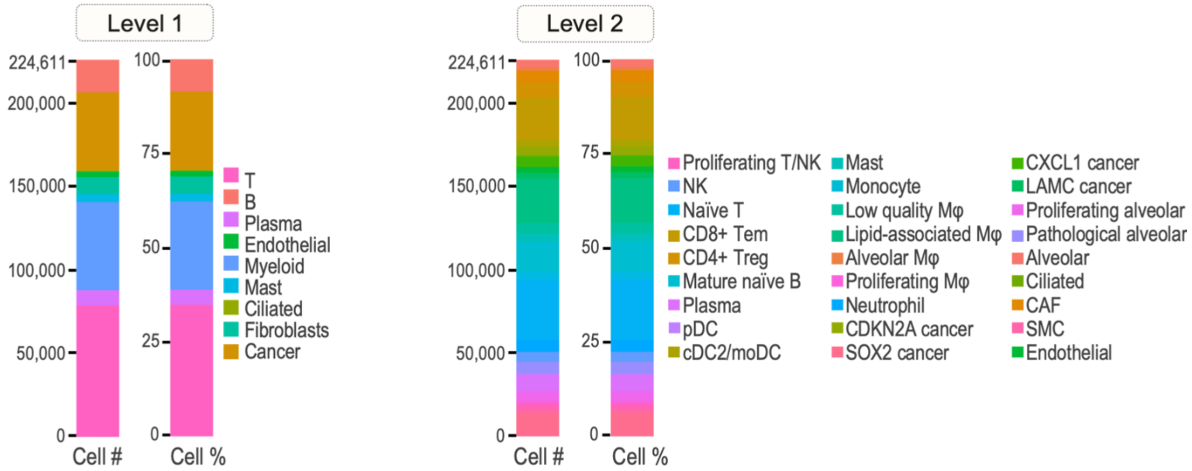

Supplementary Figure 4. Number and proportion of all cells from level 1 (left) and 2 (right) annotations.

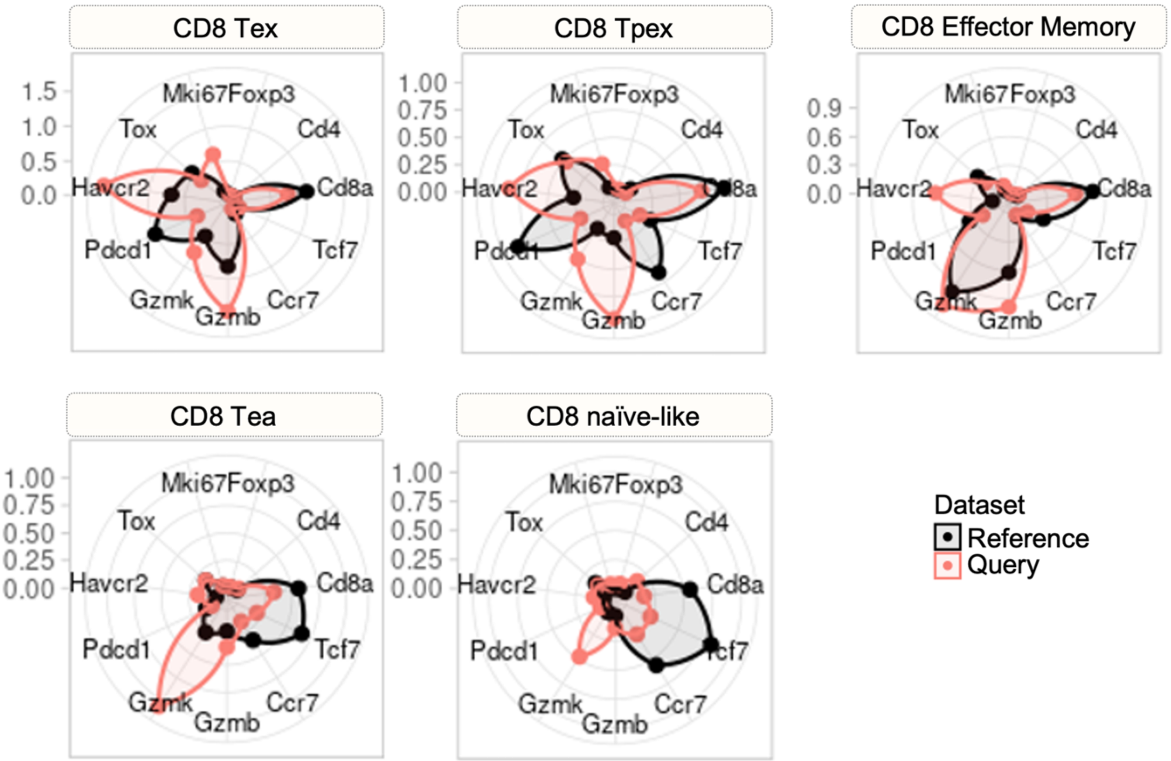

Supplementary Figure 5. Radar plots showing gene expression signature of query cells (red) and reference cells (black) of the five CD8+ T cell subtypes.
